# Blastomycosis Surveillance in 5 States, United States, 1987–2018

**DOI:** 10.3201/eid2704.204078

**Published:** 2021-04

**Authors:** Kaitlin Benedict, Suzanne Gibbons-Burgener, Anna Kocharian, Malia Ireland, Laura Rothfeldt, Natalie Christophe, Kimberly Signs, Brendan R. Jackson

**Affiliations:** Centers for Disease Control and Prevention, Atlanta, Georgia, USA (K. Benedict, B.R. Jackson);; Wisconsin Department of Health Services, Madison, Wisconsin, USA (S. Gibbons-Burgener, A. Kocharian);; Minnesota Department of Health, St. Paul, Minnesota, USA (M. Ireland);; Arkansas Department of Health, Little Rock, Arkansas, USA (L. Rothfeldt);; Louisiana Department of Health, Lafayette, Louisiana, USA (N. Christophe);; Michigan Department of Health and Human Services, Lansing, Michigan, USA (K. Signs)

**Keywords:** Blastomycosis, epidemiology, surveillance, fungi, pneumonia, respiratory diseases, United States, Arkansas, Louisiana, Michigan, Minnesota, Wisconsin

## Abstract

Blastomycosis is caused by inhalation of *Blastomyces* spp. fungi. Limited data are available on the incidence and geographic range of blastomycosis in the United States. To better characterize its epidemiologic features, we analyzed combined surveillance data from the 5 states in which blastomycosis is reportable: Arkansas, Louisiana, Michigan, Minnesota, and Wisconsin. Surveillance identified 4,441 cases during 1987–2018, a mean of 192 cases per year. The mean annual incidence was <1 case/100,000 population in most areas but >20 cases/100,000 population in some northern counties of Wisconsin. Median patient age was 46 years, 2,892 (65%) patients were male, 1,662 (57%) were hospitalized, and 278 (8%) died. The median time from symptom onset to diagnosis was 33 days. The severity of illness and diagnostic delays suggest that surveillance underestimates the true number of cases. More in-depth surveillance in additional states could elucidate blastomycosis incidence and inform efforts to increase awareness.

## Abstract 2

The median time from symptom onset to diagnosis and the severity of illness suggest that surveillance underestimates the true number of cases.

Blastomycosis is a fungal infection caused primarily by inhalation of the environmental fungi *Blastomyces dermatitidis* and *B. gilchristii*. The incubation period varies from 2 to 15 weeks, and the clinical spectrum ranges from asymptomatic to life-threatening infections involving acute respiratory distress syndrome or extrapulmonary dissemination (*1*,*2*). Most identified cases involve pulmonary infection that manifests similarly to other causes of pneumonia (*1*,*2*). The clinical similarities between blastomycosis and other pulmonary infections often result in diagnostic delays and unnecessary empiric antimicrobial drug treatment for suspected bacterial pneumonia (*3*). Because acute illnesses can self-resolve before diagnosis, and because physician awareness of this generally uncommon disease probably is low in most parts of the United States, many blastomycosis cases likely go undetected.

In the United States, most blastomycosis cases are thought to occur in the midwestern, south-central, and southeastern states, in areas surrounding the Ohio and Mississippi River valleys, the Great Lakes, and the Saint Lawrence River. Cases also occur outside these regions, indicating that the infection’s true range is broader than generally appreciated (*4*,*5*). *Blastomyces* spp. appear to have an affinity for moist soil and decomposing plant matter, but much remains unknown about its precise environmental niche (*6*,*7*). The fungus is difficult to isolate from the environment, making investigation of potential sources challenging.

Public health surveillance for blastomycosis in the United States is limited because it is currently reportable in only 5 US states: Arkansas, Louisiana, Michigan, Minnesota, and Wisconsin. Blastomycosis is not nationally notifiable, so the Centers for Disease Control and Prevention does not routinely receive case reports from states where it is reportable. Nevertheless, surveillance data represent some of the most comprehensive information about blastomycosis. Before the Council of State and Territorial Epidemiologists (CSTE) approved a standardized surveillance case definition in 2019 (*8*), state health departments used different case definitions (Appendix). However, state surveillance generally collected similar demographic, clinical, and laboratory data elements, enabling comparisons across states. We summarized available blastomycosis surveillance data to assess the overall burden of disease, geographic patterns and temporal trends, and factors associated with poor clinical outcomes.

## Methods

We combined deidentified data on blastomycosis cases reported in Arkansas during January 1995–May 2018, Louisiana during January 1987–October 2018, Michigan during January 2007–December 2017, Minnesota during January 1999–December 2018, and Wisconsin during January 1990–December 2017. We also used the Louisiana Hospital Inpatient Discharge Database to identify additional cases among hospitalized patients in Louisiana during 1999–2014.

We included data elements that were collected by >3 states. We considered event date as the earliest date associated with the case; for example, symptom onset, or first healthcare visit, laboratory test order, or public health report. We considered all laboratory tests recorded as positive for blastomycosis to be positive, even without an explicitly stated qualitative or quantitative result. Negative blastomycosis test results were not routinely available; therefore, we did not include these in the analysis.

We used patients’ state and county of residence to calculate annual state-specific incidence and county-level mean annual incidence per 100,000 persons by using yearly population estimates from the US Census Bureau, Population Division, Vintage 2015 Special Tabulation (https://www.census.gov). We used χ^2^, Fisher exact, and *t*-tests to identify factors independently associated with hospitalization or death, the Cochran-Armitage test for trends in the proportion of patients who were hospitalized or died, and negative binomial regression to assess incidence trends, and we considered p<0.05 statistically significant. We also compared demographic features and outcomes among cases associated with outbreaks (outbreak cases) and those not associated with outbreaks (nonoutbreak cases) for Minnesota and Wisconsin, the 2 states that reported outbreaks during the surveillance periods we examined. Human subjects review by the Centers for Disease Control and Prevention determined this project to be consistent with nonresearch public health surveillance.

## Results

### Descriptive Analysis

Data were available for 4,441 cases: 348 from Arkansas, 296 from Louisiana, 186 from Michigan, 671 from Minnesota, and 2,904 from Wisconsin. Most (2,892 [65%]) patients were male, and the median age was 46 years (range 0–97, interquartile range [IQR] 31–59) (Table 1). Most (64%, n = 2,778) cases were among persons of White race, 17% (740) were among persons of unknown race, 9% (406) were among persons of Black or African American races, and 5% (193) were among Asian, Native Hawaiian, or other Pacific Islander races. Most (2,828 [71%]) patients were not Hispanic or Latino; ethnicity was unknown for 1,015 (26%) patients.

**Table 1:** Patient characteristics of blastomycosis cases reported to public health, Arkansas, Louisiana, Michigan, Minnesota, and Wisconsin, USA, 1987–2018*

Characteristic	Value
Median age, y (range; IQR), n = 4,390	46 (0–97; 31–59)
Mean age, y, n = 4,390	45.3
Sex, n = 4,441	
M	2,892 (65.1)
F	1,533 (34.5)
Unknown	16 (0.4)
Race, n = 4,316	
White	2,778 (64.4)
Black or African American	406 (9.4)
Asian, Native Hawaiian, other Pacific Islander	193 (4.5)
American Indian or Alaska Native	152 (3.5)
Other or multiple races	47 (1.1)
Unknown	740 (17.2)
Ethnicity, n = 3,984	
Not Hispanic or Latino	2,828 (71.0)
Hispanic or Latino	141 (3.5)
Unknown	1,015 (25.5)
Hospitalized, n = 2,912	
Y	1,662 (57.1)
N	1,250 (42.9)
Died, n = 3,385	
Y	278 (8.2)
N	3,107 (91.8)
*Values are no. (%) except as indicated. IQR, interquartile range.	

Symptom data were available for 2,005 patients from Michigan, Minnesota, and Wisconsin beginning in 2005. The most common symptoms were cough in 79% (range by state 51%–83%) of patients, fever in 61% (range by state 38%–69%), shortness of breath in 55% (range by state 44%–85%), and weight loss in 54% (range by state 29%–62%).

Among 2,912 patients with hospitalization data, 57% (1,662) were hospitalized. The median length of hospitalization was 7 days (range 1–379 days, IQR 4–15 days; n = 1,231). Among 3,385 patients with mortality data, 278 (8%) died. The proportion of hospitalized patients did not change significantly during 2007–2017 (p = 0.252), but the proportion of patients who died increased from 9.9% to 12.4% (p = 0.017).

Data on positive blastomycosis laboratory tests were consistently available from Arkansas, Michigan, and Minnesota (Table 2). Among 1,241 reported cases from the 3 states, the most common test types were culture among 835 (67%) cases and microscopy among 333 (27%) cases. Less commonly reported tests included positive antigen tests for 206 (17%) cases and antibody tests for 59 (5%) cases.

**Table 2:** Positive laboratory tests among 1,241 blastomycosis cases reported to public health, Arkansas, Michigan, and Minnesota, United States, 1995–2018

Test type	No. (%)
Antibody	59 (4.8)
Immunodiffusion	18 (1.5)
Complement fixation	7 (0.6)
Enzyme immunoassay	30 (2.4)
Unspecified antibody test	17 (1.4)
Antigen	206 (16.6)
Confirmatory test	965 (77.8)
Culture	835 (67.3)
Microscopy*	333 (26.8)
DNA probe	40 (3.2)
PCR	2 (0.2)
Unspecified test type	166 (13.4)
Specimen type	
Culture	769 (100)
Bronchial specimen	372 (48.4)
Sputum	180 (23.4)
Other tissue besides lung	121 (15.7)
Lung tissue	21 (2.7)
Multiple specimen types	14 (1.8)
Other	61 (7.9)
Microscopy	342 (100)
Bronchoalveolar lavage	110 (32.2)
Sputum	78 (22.8)
Other tissue besides lung	52 (15.2)
Lung tissue	47 (13.7)
Multiple specimen types	24 (7.0)
Other	31 (9.1)

*Includes smear, histopathology, and unspecified microscopy tests.

Among 777 patients with available data, the median time from symptom onset to diagnosis was 33 days (range 1–2,996 days; IQR 16–75 days). We did not observe clear seasonal patterns by event month. Minnesota had 32 (5%) outbreak cases and Wisconsin had 181 (6%) outbreak cases. Outbreak- cases were more frequently among younger persons (median age 25 years) than nonoutbreak cases (median age 45 years; p = 0.0092). Outbreak cases also more often occurred among female persons (41% vs. 34% of nonoutbreak cases; p = 0.0365) and nonwhite persons (28% vs. 19% of nonoutbreak cases; p = 0.002). In addition, persons with outbreak cases were less likely to be hospitalized (45% vs. 58% of nonoutbreak cases; p = 0.003) or to have died (2% vs. 9% of nonoutbreak cases; p = 0.001).

### Bivariable Analysis

Age; female sex; non-White race; and positive antigen, culture, and microscopy tests had statistically significant associations with hospitalization (Table 3). The median age among hospitalized patients was 46 years compared with 44 years for nonhospitalized patients (p = 0.015). Female patients were more likely to be hospitalized (relative risk [RR] 1.13; 95% CI 1.06–1.21) than male patients. Persons of non-White races were more likely to be hospitalized (RR 1.13; 95% CI 1.05–1.21) than persons of White race. Patients with positive antigen tests (RR 1.25; 95% CI 1.13–1.37), positive culture (RR 1.28; 95% CI 1.20–1.36), and positive microscopy (RR 1.32; 95% CI 1.23–1.43) were more likely to be hospitalized than patients without positive results for those laboratory tests. Factors significantly associated with death were older age (median 61 years vs. 44 years; p<0.001) and positive microscopy test (RR 1.76; 95% CI 1.34–2.38).

**Table 3:** Factors associated with hospitalization or death among blastomycosis cases reported to public health, Arkansas, Louisiana, Michigan, Minnesota, and Wisconsin, United States, 1987–2018*

Characteristic	Hospitalization			Death	
	RR (95% CI)	p value		RR (95% CI)	p value
Older age	NA	0.015		NA	<0.001
Female sex	1.13 (1.05–1.21)	<0.001		1.05 (0.83–1.33)	0.681
Non-White race	1.13 (1.05–1.21)	0.002		1.08 (0.82–1.42)	0.588
Antigen test†	1.25 (1.13–1.37)	<0.001		1.27 (0.84–1.92)	0.255
Culture†	1.28 (1.20–1.36)	<0.001		1.02 (0.79–1.33)	0.864
Microscopy†	1.32 (1.23–1.43)	<0.001		1.76 (1.34–2.38)	<0.001

*NA, not applicable; RR, relative risk. †Arkansas, Michigan, and Minnesota only.

### Incidence

**Figure 1:**
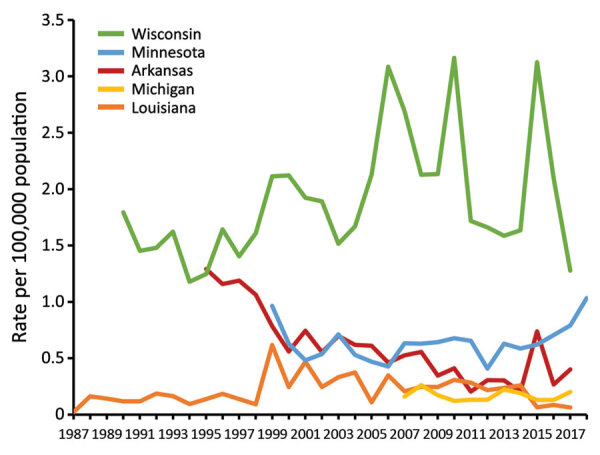
Annual state-specific incidence (no. cases/100,000 population) among 5 states in which blastomycosis is reportable, United States, 1987–2018. Cases reported during 2018 in Arkansas and Louisiana were excluded because data were not available for the entire year.

**Figure 2:**
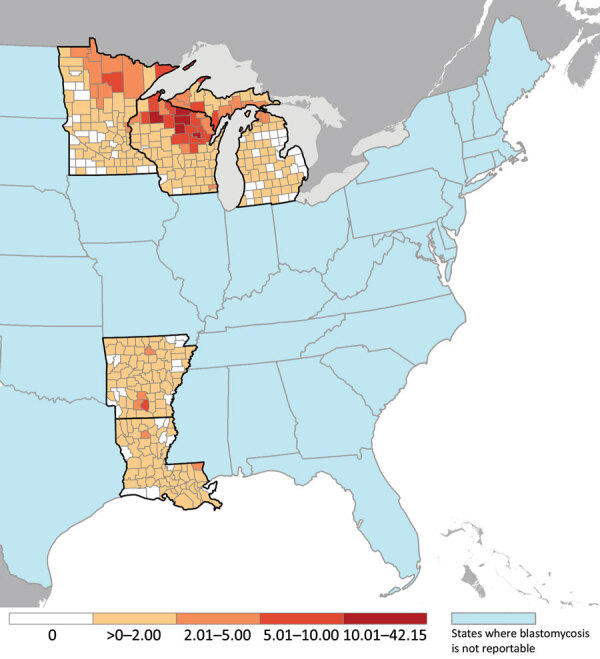
Mean annual county-specific incidence (no. cases/100,000 population) among 5 states in which blastomycosis is reportable, United States, 1987–2018. Cases reported during 2018 in Arkansas and Louisiana were excluded because data were not available for the entire year.

During years for which data were available from all 5 states, 2007–2017, surveillance detected 2,111 cases, a mean of 192 cases per year. In Arkansas, incidence declined from 1.3 cases/100,000 population in 1995 to 0.4 cases/100,000 population in 2017 (p<0.001) (Figure 1). Incidence was stable during each state’s surveillance period in Louisiana, Michigan, and Minnesota. Mean annual incidence was 0.2 cases/100,000 population in Louisiana, 0.2 cases/100,000 population in Michigan, and 0.6 cases/100,000 population in Minnesota. In Wisconsin, incidence peaked at >3 cases/100,000 population during 2006, 2010, and 2015. Mean annual county-level incidence in Wisconsin was highest in Menominee (42.1 cases/100,000 population), Lincoln (28.4 cases/100,000 population), and Vilas (26.5/100,000 population) counties (Figure 2).

## Discussion

We summarize blastomycosis surveillance data from 5 states and provide a broad update on the basic epidemiology of this enigmatic and underrecognized disease. Many patients experienced severe outcomes and diagnostic delays. Our results show that blastomycosis is underdetected, even in states where it is reportable, and that more standardized and in-depth surveillance, ideally in additional states, would help public health professionals better identify highest-risk groups and emerging areas for targeted prevention messaging.

Blastomycosis often results in severe illness, even in previously healthy persons (*9*), but this observation might be influenced by underdetection of asymptomatic or milder, self-resolving disease. The high hospitalization rate of 57% noted in this analysis demonstrates that blastomycosis surveillance detects severe cases, which is typical for passive disease surveillance. We found an annual mean of <200 cases/year; a hospitalization rate of 57% suggests that ≈110 patients are hospitalized each year from states where blastomycosis is reportable. In contrast, ≈1,000 blastomycosis-associated hospitalizations occur nationwide (*10*,*11*), showing that the limited surveillance likely underdetects cases nationally.

The average time of >1 month from symptom onset to diagnosis indicates delays in seeking healthcare, delays in diagnosis, or both. This time interval is consistent with a previous report describing a median of 23 days between examination at a healthcare facility and a median of 2.5 courses of antibacterial medications for presumed bacterial infection before pulmonary blastomycosis was correctly diagnosed (*3*). Earlier diagnosis might reduce unnecessary antibacterial drug use, time, and resources invested in searching for alternative diagnoses and could potentially improve patient outcomes. Therefore, greater public and provider education about blastomycosis is needed, especially in areas where blastomycosis is less commonly recognized.

The high proportion of patients with positive confirmatory laboratory tests, such as culture and microscopy, likely reflects detection of more severe cases because serologic tests for blastomycosis offer only presumptive evidence of infection (*12*), and serologic tests were not included in most states’ case definitions (Appendix). The associations between older age and confirmatory test types with hospitalization point to severe illness, and are unsurprising; however, why women were more likely to be hospitalized is unclear but could be related to delayed diagnosis or underdiagnosis of less severe disease in women. More blastomycosis hospitalizations typically occur among men (*10*,*13*), although a recent study found female sex was independently associated with death in blastomycosis patients with acute respiratory distress syndrome (*14*). The increased risk for hospitalization among persons of nonwhite races lends further evidence to the existence of blastomycosis-related health disparities, as previously suspected (*15*–*17*). Further studies could help determine whether these differences are related to genetic predisposition (*18*), involvement in outdoor activities resulting in exposures to *Blastomyces*, or access to medical care (*19*).

Reliance on often invasive and time-consuming tests such as culture and microscopy for diagnosis likely is a key factor in underdiagnosis of blastomycosis because these tests might not be ordered until tests for other diseases have been negative. Accordingly, most specimen types in our analysis were from bronchoalveolar lavage and lung and other tissue, which likely required biopsy. Given the prolonged time to diagnosis we identified, improved noninvasive diagnostic methods with high sensitivity and specificity for blastomycosis are needed for earlier and more frequent testing, which could prevent hospitalizations and deaths.

Consistent with previous reports, Wisconsin had the highest number of cases and incidence of the 5 states where blastomycosis is reportable, with mean annual incidence in several northern counties >20 cases/100,000 population. Peaks in incidence in Wisconsin corresponded to a known outbreak at a yard waste site in 2006 (*20*), an outbreak likely associated with multiple sources in 2010 (*21*), and an outbreak linked to recreational tubing on the Little Wolf River in 2015 (*22*). For case-patients in these outbreaks, younger age and higher likelihood of being non-White was consistent with our findings (*20*,*21*). In addition, the finding that patients with outbreak-associated cases had less severe outcomes could reflect detection of milder cases through enhanced case detection efforts during outbreak investigations. However, outbreaks comprised <6% of cases overall, suggesting that most cases occur sporadically, which also is true for histoplasmosis and coccidioidomycosis. Of note, most of northern Wisconsin is rich in soils classified as spodosols, which are characterized by high concentrations of organic matter in coarse, often sandy, particles (*23*). *Blastomyces* spp. are thought to dwell primarily in organic-rich soils. However, spodosols also occur widely in northern Michigan, where disease incidence was not elevated, and are less common in northern Minnesota, where incidence was higher. Further study, including the role of soil types, could elucidate the natural habitat of these fungi.

For most states in this analysis, the relatively stable incidence and hospitalization rates over time were consistent with a previous analysis of blastomycosis-related hospitalizations during 2000–2011 (*10*). Another study found a decline in blastomycosis-associated deaths nationwide during 1990–2010 (*15*); the reasons for the increase in deaths we observed during 2007–2017 are unclear but could reflect improvements in case follow-up, a decline in reporting of less severe cases, or other surveillance changes over time.

The limitations of our study include that pooling surveillance data based on different blastomycosis case definitions is fundamentally problematic; however, few other data sources would enable analyses of thousands of cases, which is helpful for studying this uncommon disease. Furthermore, some states’ case definitions changed over time. Although blastomycosis was reportable in each state during the years included in this analysis, Arkansas did not have a formal case definition, and Michigan did not have one until 2012. Wisconsin classified all cases as confirmed until September 2015, when their case definition changed to include confirmed and probable case classifications; for outbreaks in Wisconsin, a positive serologic blastomycosis test plus an epidemiologic link was sufficient to be considered a case. Moving forward, the standardized blastomycosis case definition from the Council of State and Territorial Epidemiologists will enable more robust comparisons between states and stratification of confirmed and probable cases.

Combining data from different times in each state is an additional potential limitation. Some states’ surveillance systems underwent changes during the analysis period; for example, data elements were added or removed, resulting in inconsistent denominators in the pooled analysis. For certain variables, such as race and ethnicity, missing data or values of “unknown” were common and demonstrate that information can be challenging to obtain because substantial time and resources often are needed to conduct case investigations (*24*). Data about environmental exposures, immunocompromised status, body site of infection, occupation, illness duration, and treatment were not available consistently from every state. Wisconsin and Minnesota conducted extensive follow-up on cases (*19*,*25*), providing deeper insight into state-specific features of blastomycosis. Collecting these types of data in a standardized way in additional states could help identify high-risk populations and activities and help inform prevention efforts.

In summary, blastomycosis remains a rarely reported but severe disease in most areas where it is under public health surveillance. Our findings indicate that blastomycosis likely is underdetected. Blastomycosis also can occur in areas outside those where it is commonly recognized (*4*) and might be emerging in new areas, such as east-central New York (*5*). Surveillance for blastomycosis in more areas and collection of more standardized, detailed data could help identify emerging geographic hotspots or clusters, new risk factors, and other epidemiologic patterns. Increased awareness among healthcare providers and the public could lead to faster diagnosis and treatment for blastomycosis patients.

Appendix State-specific blastomycosis case definitions for Arkansas, Louisiana, Michigan, Minnesota, and Wisconsin, United States.
