# Supplementary material for: Blastomycosis Surveillance in 5 States, United States, 1987–2018
Source: Emerg Infect Dis. 2021 Apr;27(4):999–1006. doi: 10.3201/eid2704.204078 (PMC8007286; doi:10.3201/eid2704.204078)
Supplement: Appendix — State-specific blastomycosis case definitions for Arkansas, Louisiana, Michigan, Minnesota, and Wisconsin, United States. [file 20-4078-Techapp-s1.pdf]

Article DOI: https://doi.org/10.3201/eid2704.204078

Blastomycosis Surveillance in 5 States, United States, 1987–2018

Appendix

State-Specific Blastomycosis Case Definitions

Arkansas

No formal case definition.

Louisiana

Blastomycosis is a fungal infection caused by *Blastomyces dermatitidis*. The organism is inhaled and typically causes an acute pulmonary infection. However, cutaneous and disseminated forms can occur, as well as asymptomatic self-limited infections.

Clinical description

Blastomyces dermatitidis causes a systemic pyogranulomatous disease called blastomycosis. Initial infection is through the lungs and is often subclinical. Hematogenous dissemination may occur, culminating in a disease with diverse manifestations.

Infection may be asymptomatic or associated with acute, chronic, or fulminant disease.

- Skin lesions can be nodular, verrucous (often mistaken for squamous cell carcinoma), or ulcerative, with minimal inflammation.
- Abscesses generally are subcutaneous cold abscesses but may occur in any organ.
- Pulmonary disease consists of a chronic pneumonia, including productive cough, hemoptysis, weight loss, and pleuritic chest pain.
- Disseminated blastomycosis usually begins with pulmonary infection and can involve the skin, bones, central nervous system, abdominal viscera, and kidneys. Intrauterine or congenital infections occur rarely.

Laboratory Criteria for Diagnosis

A confirmed case must meet at least one of the following laboratory criteria for diagnosis:

- Identification of the organism from a culture of sputum, cerebrospinal fluid (CSF), urine, or lesions
- Positive immunodiffusion test, or
- Chemiluminescent DNA probe.

Case Definition

Confirmed: A case of blastomycosis is defined as an illness characterized by clinical manifestations relating to pulmonary, cutaneous, or disseminated disease and is laboratory confirmed.

Michigan

Clinical Presentation

Blastomycosis primarily affects the lungs but can spread lymphohematogenously to extrapulmonary sites such as skin, or less commonly bone, the central nervous system and genitourinary system. The severity of the infection ranges from asymptomatic to acute or chronic pneumonia and disseminated disease, depending on the individual and factors such as age and immune system status. Elimination of the infection depends on T lymphocyte activity. Because not all of the fungal organism may be eliminated by the immune response, reactivation can occur sometimes years after the initial infection.

Signs and symptoms vary but usually include cough (possibly with blood), fever, night sweats, weight loss, chest pain, shortness of breath, muscle aches, back pain, bone pain, and fatigue. Skin lesions can be nodular to ulcerative with minimal inflammation and are most commonly located on the face and distal extremities. Symptoms may appear between 3 and 15 weeks after exposure.

Laboratory Criteria for Diagnosis*

- Isolation of *B. dermatitidis* from sputum, bronchial wash, or skin lesion OR
- Positive DNA probe performed on culture isolate OR

- Visualization of the organism in cytologic or histologic specimens by direct microscopic examination (characteristic thick walled, broad-based budding yeast)

*Note: Serologic tests (enzyme-linked immunosorbent assay, complement fixation, immunodiffusion) lack specificity and sensitivity and should not be used alone to diagnose or rule out blastomycosis. Likewise, urine antigen assays are not specific for blastomycosis and cross-reactivity occurs with histoplasmosis, paracoccidiodomycosis (South American blastomycosis), and penicilliosis.

Case classification

Confirmed: A clinically compatible illness that is laboratory confirmed.

Minnesota

A confirmed case of blastomycosis is illness in a Minnesota resident with any of the following: a positive *Blastomyces* culture, *Blastomyces* organisms visualized in tissue or body fluid, or a positive *Blastomyces* antigen test result and compatible clinical illness (e.g., cough, fever, abnormal pulmonary imaging, or skin lesions).

Wisconsin

Before September 2015

A. Clinical description: An acute or chronic illness caused by inhalation of spores of the dimorphic fungus *Blastomyces dermatitidis* that primarily affects the lungs and skin, although the clinical presentation may be variable (Appendix Table).

B. Reporting criteria: Clinical diagnosis with laboratory confirmation.

C. Laboratory criteria for confirmation:

- Isolation of *B. dermatitidis* from any sputum, bronchial washing or skin lesions, OR
- Visualization of broad-based budding yeast from an appropriate clinical specimen.

D. Wisconsin case definition: A clinically compatible illness that is laboratory confirmed.

Appendix Table. Types and clinical presentation blastomycosis case definition, Wisconsin, United States

Types of disease	Clinical presentation
Asymptomatic	May occur in about 50% of infections.
Acute pulmonary	Radiologic presentations include lobar or segmental consolidation that mimics a bacterial pneumonia. Symptoms may be highly non-specific mimicking influenza or acute bacterial infection with abrupt onset of cough, fever, chills, myalgias and arthralgias.
Chronic pulmonary	Radiologic presentations include lobar infiltrates with or without cavitation, mass mimicking bronchogenic carcinoma, or fibronodular infiltrates. This form cannot be differentiated clinically from any other form of chronic lung disease. Symptoms may be suggestive of chronic TB or histoplasmosis. Symptoms may include cough, weight loss, chest pain, night sweats, low-grade fever, skin lesions, and hemoptysis.
Skin disease	Skin lesions begin as a small papulopustular lesion that increases in size. The central part of the lesion is often encrusted. Lesions usually occur in areas of the body (particularly facial areas) exposed to the sun.
Subcutaneous nodules	Cold abscesses commonly associated with systemic manifestations. They are frequently associated with extra-pulmonary disease or multiple organ involvement.
Bone and joint infection	Seen in 10% to 40% of patients and usually lung disease present. Most commonly affect long bones, ribs, and vertebrae. Lesions are usually osteolytic and well delineated.
Genitourinary tract infection	Involvement in 10% to 30% of cases and affects the prostate, epididymis, seminal vesicle, testis, and kidney. Pain, swelling, and tenderness of the scrotum may occur.
Others	Almost any other organ can be involved, including the central nervous system, thyroid, pericardium, adrenal glands, and gastrointestinal tract.

*TB, tuberculosis

Wisconsin Case Definition Beginning in September 2015

I. Identification and Definition of Cases

A. Clinical Description: Blastomycosis is an acute or chronic illness caused by inhalation of spores of the dimorphic fungus *Blastomyces* that primarily affects the lungs and skin, although the clinical presentation and severity can be variable. Approximately 50% of infected individuals have mild symptoms or remain asymptomatic. Symptoms of acute illness may be highly non-specific, mimicking influenza or acute bacterial pneumonia with abrupt onset of cough, fever, chills, myalgia, and arthralgia. Symptoms of chronic pulmonary blastomycosis include cough, weight loss, chest pain, night sweats, low grade fever, skin lesions and hemoptysis, and may be suggestive of chronic tuberculosis, histoplasmosis, or lung cancer. A single skin lesion can indicate a localized infection resulting from dermal inoculation, but multiple skin lesions are a sign of disseminated blastomycosis. Dissemination of *Blastomyces* can occur from the lungs to almost any other organ, including the central nervous system, bones, pericardium, genitourinary tract, and gastrointestinal tract.

Criteria for a clinically compatible case includes either:

- Two or more of the following signs or symptoms:
  - Fever
  - Chest pain

    - Cough
    - Hemoptysis
    - Myalgia
    - Shortness of breath
    - Headache

OR

- One or more of the following clinical findings:
    - Single skin lesion
    - Abnormal chest imaging (e.g., pulmonary infiltrates, cavitation, enlarged hilar or mediastinal lymph nodes, pleural effusion)
    - Clinical evidence of disseminated disease (one or more of the following):
        - Multiple skin lesions
        - Peripheral lymphadenopathy
        - Bone involvement
        - Pancytopenia, as evidence of bone marrow involvement
        - Enlargement of the liver, spleen, or abdominal lymph nodes
        - Meningitis, encephalitis, or focal brain lesion

B. Laboratory Criteria

- Confirmatory laboratory criteria (one or more of the following):
    - Culture of *Blastomyces* from a clinical specimen
    - Identification of characteristic *Blastomyces* large, broad-based, budding yeast in tissue or sterile body fluid by histopathology
    - Demonstration of *Blastomyces*-specific nucleic acid in a clinical specimen using a validated assay (i.e., PCR)
- Supportive laboratory criteria:

- o Identification of characteristic *Blastomyces* large, broad-based, budding yeast in tissue or clinical body fluid (e.g., CSF, sputum, BAL, aspirate) by cytopathology, or
- o ≥4-fold rise in *Blastomyces* serum immunodiffusion antibody titers taken at least 2 weeks apart, or
- o Detection of quantifiable *Blastomyces* antigen in serum, urine, or other body fluid by an enzyme immunoassay test;

AND

- o No compelling laboratory evidence of another mycotic infection is available

C. Wisconsin Surveillance Case Definition

Confirmed: A clinically compatible case that meets at least one of the confirmatory laboratory criteria.

Probable:*

- A clinically compatible case that meets supportive laboratory criteria; or
- A clinically compatible case that does not meet laboratory criteria but is epidemiologically linked to a confirmed case.

*Note: Illness in a person with compelling laboratory evidence (e.g., culture, histopathology, seroconversion) of a different fungal infection, such as histoplasmosis or coccidioidomycosis, and meeting only supportive laboratory criteria for blastomycosis should not be counted as a case of blastomycosis because other fungal infections can cause false positive *Blastomyces* antigen and antibody test results.
